# Supplementary material for: Studies of the Efficacy of Low-Dose Apatinib Monotherapy as Third-Line Treatment in Patients with Metastatic Colorectal Cancer and Apatinib’s Novel Anticancer Effect by Inhibiting Tumor-Derived Exosome Secretion
Source: Cancers (Basel). 2022 May 19;14(10):2492. doi: 10.3390/cancers14102492 (PMC9139438; doi:10.3390/cancers14102492)
Supplement: Supplementary file 1 [file cancers-14-02492-s001.zip › cancers-1684975-supplementary.pdf]

Table S1. Sequences of primers for real-time PCR.

Gene	Primers-Forward	Primers-Reverse
GAPDH	5′-TGCACCACCAACTGCTTAGC -3′	5′-GGCATGGACTGTGGTCATGAG -3′
AP3S2	5′-GATTGAGGCTCAAAACAGGCTG G-3′	5′-CAAACTGGGACAGGTTGGGAAC -3′
YPEL4	5′-CACGATGAGCTTATTTCCAAGTC C -3′	5′-CCAGTGTGGTTTTGCAGCTCTC -3′
SULT1A3	5′-ACGGCTCATCAAGTCACACCTG -3′	5′-TGCGCCTTTTCCATACGGTGGA -3′
TCEAL7	5′-TGAACGCCAGCAAACAGAAGGG -3′	5′-CACCTTTCCATCTCATCTCCCTC -3′
TRIM59	5′GAAGGAAATACGAGAGCAGCAG C -3′	5′-GGTTGAACCTCAGGAAGTGGTC -3′
CCR4	5′-CTCTGGCTTTTGTTCACTGCTGC -3′	5′-AGCCCACAGTATTGGCAGAGCA -3′
EEA1	5′- CTTCTAGCCACCAGGCAAGATC -3′	5′-CCAATGTAGCCTTGGCAGTCTTC -3′
LAMP2	5′-GGCAATGATACTTGTCTGCTGC -3′	5′-GTAGAGCAGTGTGAGAACGGA-3′
Rab11a	5′-AGCACCATTGGAGTAGAGTTTG C-3′	5′AAGGCACCTACAGCTCCACGAT-3′
Rab11b	5′-TGTCGGTGCCTCACTGGATTGA-3′	5′-TGTCATAGTGCTCAGCCAGGCT-3′
Rab11c	5′-ACTGCTCTTCCTGGAGACCTCA-3′	5′-GCTGTTCTGTCTCTGCTTGGAC-3′
Rab27a	5′-GAAGCCATAGCACTCGCAGAGA -3′	5′-CAGGACTTGTCCACACACCGTT-3′
Rab27b	5′-TGGCAACAAGGCAGACCTACCA -3′	5′-CTCCACATTCTGTCCAGTTGCTG-3′
Snap23	5′-GGTGACAAATGGTCAGCTTCAG C-3′	5′-CCAGGATACTGCCCACTTGAGT-3′
Sytaxin4	5′-CGGCAGACTATTGTCAAACTGG G-3′	5′-ATCTCATCGCGCAGGTTCTGCA-3′
VAMP2	5′CTCCAAACCTCACCAGTAACAGG -3′	5′-AGCTCCGACAGCTTVTGGTCTC-3′

Fig.4F

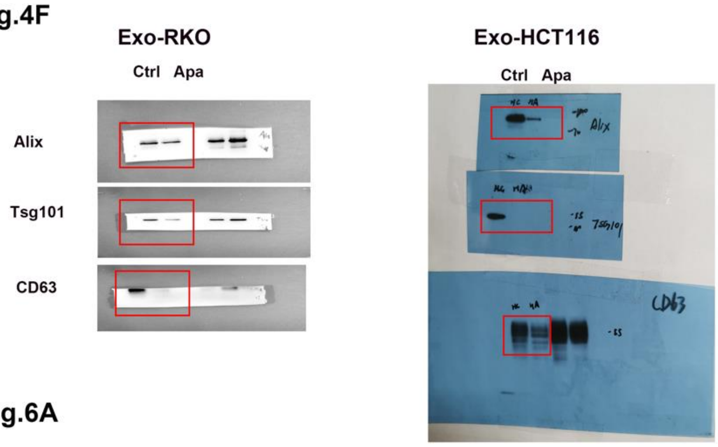

Fig.6A

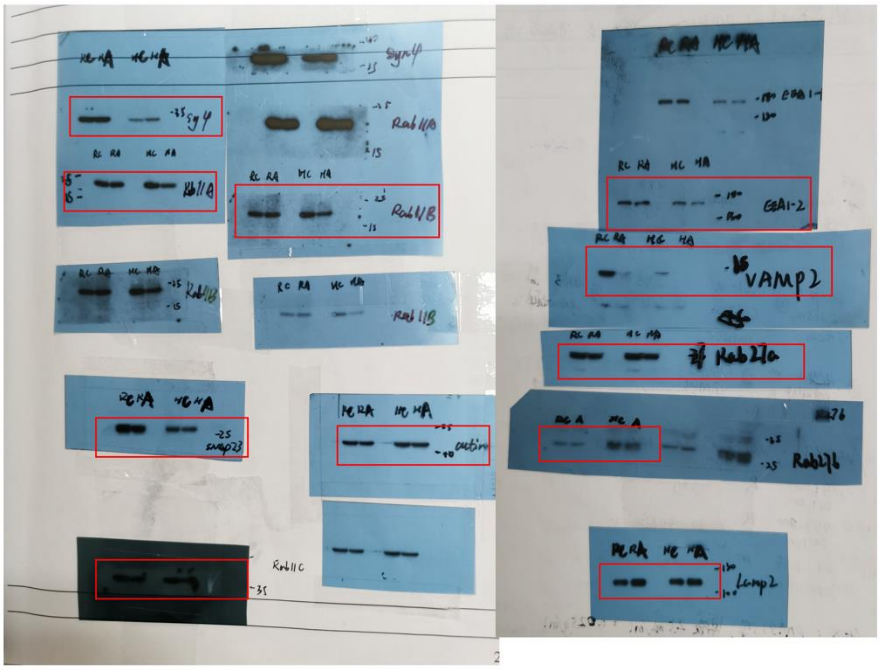

Figure S1. Original blots of western blot analyses in this paper (Fig.4F (machine reading blots) and Fig.6A (original films).
